# Skin and soft tissue infections and current antimicrobial prescribing practices in Australian aged care residents

**DOI:** 10.1017/S0950268819000128

**Published:** 2019-02-22

**Authors:** N. J. Bennett, N. Imam, R. J. Ingram, R. S. James, K. L. Buising, A. L. Bull, C. S. Chen, K. A. Thursky, L. J. Worth

**Affiliations:** 1 National Centre for Antimicrobial Stewardship, 792 Elizabeth St, Melbourne, Vic. 3000, Australia; 2 Victorian Healthcare Associated Infection Surveillance System Co-ordinating Centre, 792 Elizabeth St, Melbourne, Vic. 3000, Australia; 3 Department of Medicine, The University of Melbourne, Parkville, Vic. 3010, Australia

**Keywords:** Aged care, antimicrobial drugs, skin infections, surveillance system

## Abstract

To determine the burden of skin and soft tissue infections (SSTI), the nature of antimicrobial prescribing and factors contributing to inappropriate prescribing for SSTIs in Australian aged care facilities, SSTI and antimicrobial prescribing data were collected via a standardised national survey. The proportion of residents prescribed ⩾1 antimicrobial for presumed SSTI and the proportion whose infections met McGeer *et al.* surveillance definitions were determined. Antimicrobial choice was compared to national prescribing guidelines and prescription duration analysed using a negative binomial mixed-effects regression model. Of 12 319 surveyed residents, 452 (3.7%) were prescribed an antimicrobial for a SSTI and 29% of these residents had confirmed infection. Topical clotrimazole was most frequently prescribed, often for unspecified indications. Where an indication was documented, antimicrobial choice was generally aligned with recommendations. Duration of prescribing (in days) was associated with use of an agent for prophylaxis (rate ratio (RR) 1.63, 95% confidence interval (CI) 1.08–2.52), PRN orders (RR 2.10, 95% CI 1.42–3.11) and prescription of a topical agent (RR 1.47, 95% CI 1.08–2.02), while documentation of a review or stop date was associated with reduced duration of prescribing (RR 0.33, 95% CI 0.25–0.43). Antimicrobial prescribing for SSTI is frequent in aged care facilities in Australia. Methods to enhance appropriate prescribing, including clinician documentation, are required.

## Introduction

In Australia, approximately 2670 aged-care homes (ACHs) provide resident accommodation and support, including assistance with day-to-day living and intensive forms of care. Almost 180 multi-purpose services (MPSs) also deliver a flexible mix of acute, sub-acute and aged care services to best meet a community's needs [1].

In these facilities, elderly populations are at increased risk for skin and soft tissue infections (SSTIs) [2] related to physiological, aged-related changes to the skin, decline in immune function and the presence of comorbid conditions such as vascular disease [3, 4]. It is important that antimicrobial therapy for SSTIs is targeted and appropriately prescribed, in order to improve clinical outcomes and reduce risks of adverse outcomes (e.g. *Clostridium difficile* infection) and development of antimicrobial resistance associated with improper or prolonged use [5, 6].

Since 2015, all Australian aged care facilities (ACHs and MPSs) have been invited annually to participate in the Aged Care National Antimicrobial Prescribing Survey (acNAPS). This structured point-prevalence survey enables prevalence of infections and antimicrobial prescribing for all residents in these facilities who are present on the survey day to be estimated. Findings to date have identified SSTIs as the second most common infection among residents [7].

The objectives of this study were to: (i) determine the relative burden of specific SSTIs, (ii) estimate the prevalence and nature of antimicrobial prescribing for SSTIs and (iii) evaluate factors contributing to inappropriate prescribing of antimicrobial agents for SSTIs in Australian aged care facilities participating in the acNAPS.

## Methods

### Survey methodology

Point-prevalence surveys of infections and antimicrobial use were completed by nurses, infection control professionals or pharmacists at participating facilities on a single survey day between 19 June and 1 September 2017. All data were obtained by review of residents’ records (medical, nursing and medication charts).

Data collected about antimicrobials prescribed on the survey day included clinical indication (reason for commencing an antimicrobial agent), rationale (i.e. prophylaxis *vs.* therapeutic), route, frequency and duration (start and review/stop dates). If not documented, the clinical indication for commencing the antimicrobial was interpreted by the surveyor based on other information available in the clinical records. If the antimicrobial start date was known and <6 months prior to the survey day, any documented infection signs and/or symptoms on the antimicrobial start date or 6 days prior were reported.

Prior to the survey day, education regarding the surveillance methodology was provided. Surveyors could participate in training webinars and a detailed user manual could be accessed via the National Antimicrobial Prescribing Survey (NAPS) website. Individual support was available by telephone or email liaison with the NAPS staff. Surveys were submitted on-line via a secure web portal.

### Definitions

Evaluable SSTI clinical indications for prescribing antimicrobials were mapped to McGeer *et al.* standardised and accepted surveillance definitions [8] and included: cellulitis, soft tissue and wound infection; fungal skin infection; herpesvirus skin infection; and scabies. In accordance with the McGeer ‘skin, soft tissue and mucosal infection’ definition, conjunctivitis and oral candidiasis were also included. If infection signs and/or symptoms were reported, ‘possible’ infections were defined as those where at least one element of McGeer *et al.* criteria was reported. ‘Confirmed’ infections were defined as those that met all elements required for McGeer *et al.* criteria (Table 1).

**Table 1.** Definitions for infections in surveyed aged care residents (McGeer *et al.* [1])

No.	Infection	Confirmed infection	Criterion
A	Cellulitis/soft tissue/wound	At least one criterion	Pus present at wound skin or soft tissue site At least four sub-criteria Heat Redness Swelling Tenderness or pain Serous discharge One constitutional criteria^a^
B	Conjunctivitis	At least one criterion	Pus from one or both eyes present >24 h New or increased conjunctival redness Itching or pain >24 h
C	Fungal skin infection	Criterion 1 and 2	Characteristic rash or lesions Doctor or laboratory confirmation
D	Herpesvirus skin infection	Criterion 1 and 2	Vesicular rash Doctor or laboratory confirmation
E	Oral candidiasis	Criterion 1 and 2	Presence of raised white patches or plaques in mouth Doctor or dental provider confirmation
F	Scabies	Criterion 1 and 2	Maculopapular and/or itching rash At least one sub-criteria Doctor or laboratory confirmation Linkage to a laboratory-confirmed case of scabies

a Constitutional criteria include fever, leucocytosis, acute change in mental status from baseline and acute functional decline.

Antimicrobial prescriptions included all antibiotic, antiviral, antifungal and anti-parasitic agents. The prevalence of antimicrobial prescribing for SSTI was defined as the proportion of all residents that were prescribed at least one antimicrobial for a SSTI clinical indication on the survey day.

### Data analysis

The prevalence of residents prescribed ⩾1 antimicrobial for all clinical indications and the subset of indications specifically related to SSTI were calculated. For residents prescribed at least one antimicrobial where the known start date was <6 months prior to the survey day, the proportion of those with at least one infection sign and/or symptom and those with a McGeer *et al.* confirmed infection was also determined.

The frequency of specific antimicrobial agents for each SSTI clinical indication was calculated. Additionally, and as a measure of appropriateness, the prescribed agents were compared to the choice and route of antimicrobials recommended in national prescribing guidelines [9, 10] for mild early cellulitis or wound infection, cutaneous candidiasis, conjunctivitis, oral candidiasis, herpes simplex infections and scabies (including crusted scabies) (Supplementary Appendix I). This analysis was stratified according to route (oral or topical).

For prescriptions with known start dates <6 months prior to the survey day, duration of prescription (in days) was analysed using mixed-effects regression models. With a Poisson distribution for the dependent variable, significant over-dispersion was seen (sum of squared Pearson residuals *vs.* residual degrees of freedom, *P* < 0.01); test for zero-inflation was insignificant (expected *vs.* observed number of zeroes, *P* = 0.39). Therefore, a negative binomial distribution was used.

Mixed-effects negative binomial modelling was performed by inclusion of patient (age, sex, known allergies, hospitalisation in last 30 days) and prescription-level covariates (antimicrobial type, indication specification, frequency specification, mode (written or phone), indication documentation, review/stop date recorded and prophylactic or treatment) at level 1 variables. Each participating ACH was added to level 2 as a random intercept to account for intra-facility variability. Starting with a maximal model containing all relevant covariates, model selection was performed using likelihood ratio tests. Model fit was assessed using residual plots. All analyses were conducted in the R programming language (Version 3.3.2).

### Ethics

The study was reviewed and approved by the Melbourne Health Human Research Ethics committee as a Quality Assurance/Negligible Risk Research project (QA 201 3066).

## Results

Of the 292 participating aged care facilities, 67.8% were located in Victoria and 68.8% were operated by the Victorian State Government. Of the total 12 307 residents audited, 67.0% were female and the median age was 87.3 years (interquartile range (IQR): 80.7–92.0). A total of 1087 residents were prescribed an antimicrobial agent (8.8%), 452 specifically for a SSTI clinical indication (3.7%).

Four hundred and eighty-four antimicrobials were prescribed for a SSTI clinical indication. These antimicrobials were mostly administered topically (70.0%) or orally (29.6%). About two-thirds (65.7%) had a known start date that was <6 months prior to the survey day – the median duration for these prescriptions was 7.0 days (IQR: 3.5–29.0) up to the survey day.

The most frequently reported clinical indications among residents were cellulitis, soft tissue or wound infection (*n* = 130), followed by fungal skin infection (*n* = 60), conjunctivitis (*n* = 49) and oral candidiasis (*n* = 18). Herpes simplex or zoster infections were reported in three instances and scabies in one instance. Table 2 summarises the prevalence of SSTI indications.

**Table 2.** Prevalence of antimicrobial prescribing for skin, soft tissue and mucosal clinical indications and proportion of confirmed infections

Clinical indication for commencing antimicrobial	Residents with prescription prevalence (%) (*n* = 12 307 residents)	Residents with prescriptions that the start date was <6 months		
		Total number	At least one infection sign + /or symptom (%)	McGeer *et al* confirmed infection (%)
Cellulitis, soft tissue or wound infection	130 (1.06)	121	82 (67.77)	48 (39.67)
Fungal skin infection	60 (0.49)	41	20 (48.78)	9 (21.95)
Conjunctivitis	49 (0.40)	37	27 (72.97)	27 (72.97)
Oral candidiasis	18 (0.15)	16	8 (50.00)	5 (31.25)
Herpes simplex or zoster	3 (0.02)	3	2 (66.67)	2 (66.67)
Scabies	1 (0.01)	1	0 (0.00)	0 (0.00)
Other	210 (1.71)	95	–	–
Total skin, soft tissue and mucosal^a^	452 (3.67)	314	139 (44.27)	91 (28.98)

a Some residents prescribed antimicrobial therapy for >1 clinical indication.

Where an antimicrobial agent was prescribed for an SSTI clinical indication and the known start date was <6 months prior to the survey day, a confirmed infection was infrequently documented (29.0%). For antimicrobial prescriptions for conjunctivitis, infections confirmed by McGeer *et al.* criteria were present in 73.0%; for cellulitis, soft tissue or wound infections, infection confirmed by McGeer *et al.* criteria was present in 40.0% (Table 2).

For cellulitis, soft tissue and wound infections, cephalexin and flucloxacillin were prescribed most frequently (*n* = 54 (40.0%) and 18 (13.3%), respectively). For fungal skin infection, clotrimazole and miconazole were most frequently prescribed (*n* = 37 (59.7%) and 14 (22.6%), respectively). Topical chloramphenicol was the most frequently prescribed agent for conjunctivitis (*n* = 45 (90.0%)). Overall, clotrimazole was the most frequently prescribed agent (39.6%), usually for unspecified clinical indications (69.3%).

**Fig. 1.**
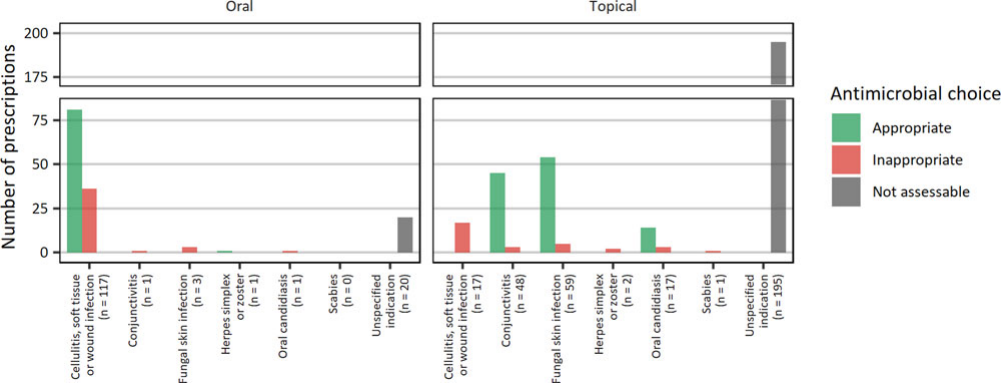
Appropriateness of antimicrobial choice by skin indications.

Where a specific clinical indication for prescribing oral or topical antimicrobial agents was known, 73.0% were aligned with national recommendations for antimicrobial prescribing. For cellulitis, soft tissue or wound infections, fungal skin infections, conjunctivitis and oral candidiasis, antimicrobial choice and route was appropriate in 60.0%, 87.1%, 91.8% and 77.8%, respectively (Fig. 1). For cellulitis, soft tissue or wound infections, where selection of an agent was assessed as inappropriate, doxycycline (*n* = 8) and trimethoprim/sulfamethoxazole (*n* = 6) were most frequently prescribed. For 215 prescriptions, specific indications were not known, and appropriateness of agent selection was unable to be determined.

Duration of prescribing (in days) was significantly associated with use of an agent for prophylaxis (rate ratio (RR) 1.63, 95% confidence interval (95%CI) 1.08–2.52), PRN (*pro re nata* or as required) orders (RR 2.10, 95% CI 1.42–3.11) and prescription of a topical agent (RR 1.47, 95% CI 1.08–2.02). Documentation of a review or stop date was associated with reduced duration of prescribing (RR 0.33, 95% CI 0.25–0.43). Results of regression modelling are summarised in Table 3. Intra-facility variability accounted for 53.5% (95% CI 53.3–53.6%) of all variability in duration of prescribing.

**Table 3.** Factors associated with duration (in days) of antimicrobial prescribing in aged care residents (*n* = 317)^a^

	*n*	Rate ratio	95% CI
Gender			
Male	105	1.09	0.84–1.42
Female	212	Referent	
Allergies to antimicrobial agent(s)			
Not documented	6	1.14	0.41–3.33
Yes	68	0.75	0.56–1.03
No	243	Referent	
Indication documented by clinician?			
Yes	273	0.75	0.52–1.08
No	44	Referent	
Specific indication observed by surveyor?			
Yes	222	0.99	0.73–1.33
No	95	Referent	
Agent administered as prophylactic?			
Yes	29	1.63	1.08–2.52
No	288	Referent	
Type of antimicrobial			
Antibacterial	189	0.73	0.54–0.98
Antiviral/anti-parasitic	4	1.17	0.44–3.83
Antifungal	124	Referent	
Route of administration			
Topical	174	1.47	1.08–2.02
Non-topical	143	Referent	
Frequency of administration			
PRN (as required)	41	2.10	1.42–3.11
Specific	276	Referent	
Review/stop date recorded?			
Yes	173	0.33	0.25–0.43
No	144	Referent	

a Antimicrobial prescriptions with known start dates <6 months only included.

## Discussion

Our study has provided a detailed evaluation of prescribing patterns for SSTIs in Australian aged care residents. We identified topical antifungal therapy to be the most frequently prescribed agent, and *β*-lactam agents to be most frequently prescribed oral agents for skin soft tissue and wound infections. A number of clinical practices were identified to be associated with longer duration of prescribing, including use of an agent for prophylaxis, PRN orders and prescription of topical agents.

Evaluation of the quality of prescribing included comparison of antimicrobial choice and route with national prescribing guidelines, and analysis of factors contributing significantly to prolonged prescriptions. We observed the majority (73.0%) of targeted prescribing to be in accordance with national guidelines for antimicrobial therapy. Similarly, a European study of nursing homes found 76% of antibiotics prescribed for SSTIs were compliant with national guidelines [11]. However, a large proportion of prescribed agents were for unspecified clinical indications, meaning that appropriateness of antimicrobial agents could not be assessed.

We identified duration of prescribing of antimicrobial agents to be significantly associated with use of an agent for prophylaxis, PRN orders and prescription of a topical agent. This is consistent with international clinical observed practice, whereby prophylaxis may be commenced, but the duration unknown or undefined [12]. While PRN orders are likely to be applied to topical (rather than oral) antimicrobial therapies, it is conceivable that these would be less likely to be reviewed by clinicians and ceased when no longer clinically indicated. Conversely, documentation of a review or stop date was associated with reduced duration of prescribing. This finding supports the beneficial role of improved documentation to reduce antimicrobial treatment courses, potentially reducing risks of developing antimicrobial resistance.

For the current study, prescribing practices were compared to national prescribing guidelines, and analysis of factors contributing significantly to prolonged prescriptions was performed. While these have not been specifically validated as measures of quality in aged care, we believe that comparison with a gold standard does enable benchmarking, and that risks for prolonged prescribing are an important consideration for potentially reducing unnecessary exposure to antimicrobial agents. Looking ahead, we acknowledge that an assessment tool for appropriateness is required, and that this should also incorporate understanding of allergies to antimicrobial agents, as well as local guidelines within aged care facilities.

Being reliant upon facility-level point-prevalence surveys, our study is limited by potential variability in case-mix of populations, varied quality of captured data and the chosen reporting period, which may all impact upon generalisability of results. Nonetheless, we believe this method to be advantageous for surveillance in aged care settings, given the relative ease of conducting point-prevalence surveys and minimal requirement for resources when compared to incidence surveys, which require more detailed data concerning admissions or discharges to a facility, continuous monitoring and therefore increased resources [13].

Our study is also limited by the representativeness of the surveyed population, mostly Victorian public aged care facilities. In Australia, aged care facilities are located in all states or territories, mainly New South Wales and are largely owned by not-for-profit or private organisations [1]. Further, the audit tool requires review of resident records to confirm the clinical indication and presence of signs and/or symptoms for infections. The quality of these data is dependent upon completeness of documentation. It is also unknown if the prevalence of residents prescribed at least one antimicrobial would have been lower if any PRN orders never administered had been excluded from the analyses.

In conclusion, we observed cellulitis, soft tissue or wound infections to be the most frequently reported SSTI in residents of Australian aged care facilities. While targeted antimicrobial prescribing is generally aligned with national recommendations, we observed a large proportion of prescribed antimicrobial agents to be for unspecified clinical indications. Interventions for improved antimicrobial prescribing should include review of prescribing of agents for prophylaxis, PRN orders and prescribing of topical agents. Methods to enhance clinician documentation of indications for antimicrobial therapy are required, and development of standardised tools for assessing appropriateness of antimicrobial agents in elderly populations would support these interventions.

## Supplementary material

For supplementary material accompanying this paper visit https://doi.org/10.1017/S0950268819000128. click here to view supplementary material
